# Knowledge translation dataset: An e-health intervention for pregnancy in inflammatory bowel disease

**DOI:** 10.1016/j.dib.2018.12.085

**Published:** 2018-12-30

**Authors:** Reed T. Sutton, Kelsey Wierstra, Vivian W. Huang

**Affiliations:** a Department of Medicine, University of Alberta, Edmonton, Canada; b Department of Medicine, University of Toronto, Toronto, Canada

**Keywords:** Inflammatory bowel disease, Pregnancy, Maternal and child health, Sexual and reproductive health, Concerns, CCPKnow, Medications, Beliefs about medications, BMQ, Medication adherence, MARS

## Abstract

This article presents data collected from a cohort of patients with inflammatory bowel disease, who expressed interest in family planning and reproductive health in their clinical context. They were randomized (1:1, text-only vs. multimedia content) to access an online e-health portal containing educational information on the topic. The data collected includes baseline demographics, medication history, reproductive history, as well as standardized, validated questionnaires on knowledge (‘CCPKnow’), reproductive concerns, beliefs about medications (‘BMQ’), and medication adherence (‘MARS-5’). These questionnaires were administered prior to the intervention, immediately after accessing the materials, and a minimum of 6 months later (without re-accessing the online material). Two publications have been generated from analysis and aggregation of the CCPKnow data (“Pregnancy-related Beliefs and Concerns of Inflammatory Bowel Disease Patients are Modified After Accessing e-Health Portal” (Sutton et al., in press), “Innovative Online Educational Portal Improves Disease-Specific Reproductive Knowledge Among Patients With Inflammatory Bowel Disease” (Sutton et al., 2018) however this is an extensive dataset that could be analyzed or combined with others’ datasets for further insights.

**Specifications table**

**Table:** 

Subject area	Medicine and dentistry
More specific subject area	1. Inflammatory Bowel Disease 2. Reproductive Health
Type of data	Table, Figures, Raw Spreadsheet
How data was acquired	Online questionnaires + Website Analytics (WordPress)
Data format	Raw and Tabulated
Experimental factors	No pre-treatment of data.
Experimental features	Randomization to text-only or multimedia e-health portal. Pre-, post-, 6+months post-intervention questionnaires.
Data source location	Alberta, Canada (Primarily Edmonton, 53.5444°N, 113.4909°W)
Data accessibility	Dataset published on Mendeley Data: https://data.mendeley.com/datasets/g223h3p8gy/1
Related research articles	• Sutton R, Wierstra K, Bal J, et al. Pregnancy-related Beliefs and Concerns of Inflammatory Bowel Disease Patients are Modified After Accessing e-Health Portal. *Patient Education and Counceling.* 2019; (in press) [1]. • Wierstra K, Sutton R, Bal J, et al. Innovative Online Educational Portal Improves Disease-Specific Reproductive Knowledge Among Patients With Inflammatory Bowel Disease. *Inflamm Bowel Dis*. 2018;24(12):2483–2493. doi:10.1093/ibd/izy1612.

**Value of the data**

• CCPKnow, MARS-5, and BMQ are validated questionnaires, and so this data provides a benchmark for comparison of patient populations from other centers, or contrast to populations from different demographics.
• The data is extensive, containing disease history and patient demographics, information on user׳s technology preferences, methods of learning, and more.
• These data can be utilized by researchers with interest in preconception and pregnancy in inflammatory bowel disease patient populations, combined with others’ datasets, and analyzed for further insights.

## 1 Data

The data collected at baseline includes demographics, DOB (converted to birth year to protect PII), highest level of education, employment, income, family history, languages, marital status, and extensive reproductive history including children, current pregnancy, pregnancy outcomes and health, future and current family plans. Inflammatory Bowel Disease history was also collected including family history of IBD, diagnosis, year diagnosed, previous and current medications, specialist access, discussion of reproductive topics in IBD, and sources of pregnancy in IBD information accessed. All of this data is included in Appendix A.

Standardized, validated questionnaires on knowledge (‘CCPKnow’), reproductive concerns, beliefs about medications (‘BMQ’), and medication adherence (‘MARS-5’) were also collected at pre-intervention, post-intervention, and 6+ months later. They are detailed below and included in Appendix B.

1. *Patient reproductive concerns:* Six IBD-specific reproductive concern questions were asked (adapted from Marri et al.) [3], [4]. Participants responded ‘yes’ or ‘no’ to each statement.
2. *MARS-5:* Self-reported adherence assessment utilizing a 5 statement questionnaire evaluating non-adherent medication taking behaviors [5]. Each statement is scored on a 5-point Likert scale, ranging from 1 = always to 5 = never.
3. *BMQ IBD S18:* The validated BMQ questionnaire, a version specific to IBD, was used to measure beliefs that influenced adherence to medications. Questions from the BMQ are classified as “specific”(personal beliefs), further subdivided into *necessity* and *concerns* scales [6]. Participants ranked statements from each scale on a Likert spectrum (1 = strongly disagree, 5 = strongly agree). This particular version of the BMQ included 8 *necessity* statements and 9 *concerns* statements.
4. *CCPKnow*: 17 item validated score used to measure IBD-specific reproductive knowledge [7]. Correct answers to the questions (5 options each question) are usually summed to form a total score, and typically categorized into levels as follows;
  i. poor (0–7),
  ii. adequate (8–10)
  iii. good (11–13)
  iv. very good (14–17)

Feedback questions were asked regarding the intervention itself at post-intervention and 6 months later. The questions are included in Appendix C. Finally, analytics data on the usage and access time for specific pages and users was pulled from the portal, and is included in the raw datasets.

## 2 Experimental design, materials, and methods

### 2.1 Design

A prospective randomized intervention study was conducted. Patients were invited to access an online e-health portal for reproductive health information in the context of inflammatory bowel disease. Recruitment methods have been described [1], [2]. Upon enrollment, patients completed the pre-intervention questionnaire (Appendices A and B), and were randomized (1:1) to receive access to either a text-only version of the portal content, or a multimedia version containing the same text content supplemented by videos, animated diagrams, slideshow, and self-testing quizzes. Patients were given 60 day access to the portal׳s content, before completing the post-intervention questionnaire (Appendices B and C). Six months later they completed the same questionnaires again (Appendices B and C).

### 2.2 Educational content

The portal׳s education content (the ‘intervention’) was drafted by expert systematic literature review on topics previously found to be of interest to this patient population [4]. Literature up to May 2014 was included. The content was aggregated into five modules:

1. I have IBD, can I become pregnant?
  (http://pregnancy.ibdclinic.ca/information/ibd-can-i-get-pregnant/)
2. I have IBD, will my child have IBD?
  (http://pregnancy.ibdclinic.ca/information/will-my-child-have-ibd/)
3. I have IBD, could surgery affect my ability to become pregnant?
  (http://pregnancy.ibdclinic.ca/information/ibd-surgery-affect-ability-become-pregnant/)
4. I have IBD, how does IBD affect pregnancy?
  (http://pregnancy.ibdclinic.ca/information/how-does-ibd-affect-pregnancy/)
5. I have IBD, how does IBD affect delivery, postpartum, and breastfeeding?
  (http://pregnancy.ibdclinic.ca/information/ibd-affect-delivery-postpartum-breastfeeding/)?

**Please note that the content at the above links may have been updated since being used in the described study.

### 2.3 Setting, participant characteristics

Described previously, participants were 18–45 year old male and female IBD patients known to the IBD Clinic (University of Alberta Hospital, Edmonton, AB, Canada). The IBD Clinic serves patients from all over Alberta, and from surrounding provinces. The demographics have been tabulated for participants completing each of the three study time points (Table 1).

**Table 1:** Demographics and medical information for participants at three study time points, pre-intervention, post-intervention, and 6+ months post-intervention.

**Category**	**Pre-intervention Completers**	**Post-intervention Completers**	**6-month study completers**
	**(n = 101)**	**(n = 78)**	**(n = 37)**
	**No. (% of total)**	**No. (% of total)**	**No. (% of total)**
Age at prestudy, y			
18–24	24 (23.8)	15 (19.2)	7 (18.9)
25–29	32 (31.7)	29 (37.2)	11 (29.7)
30–34	28 (27.7)	21 (26.9)	12 (32.4)
35–39	12 (11.9)	10 (12.8)	5 (13.5)
40–45	5 (5.0)	3 (3.8)	2 (5.4)

Sex			
Male	18 (17.8)	15 (19.2)	7 (18.9)
Female	83 (82.2)	63 (80.8)	30 (81.1)

Marital status			
Single, never married	33 (32.7)	22 (28.2)	14 (37.8)
Divorced	3 (3.0)	3 (3.8)	0 (0)
Partnered	65 (64.4)	53 (67.9)	23 (62.2)

1st language			
English	92 (91.1)	71 (91.0)	32 (86.5)

Income (n = 77)			
Less than $20, 000	11 (11.2)	8 (10.4)	5 (13.5)
$20,000 to $39,999	6 (6.1)	3 (3.9)	1 (2.7)
$40,000 to $69,999	25 (25.5)	23 (29.9)	11 (29.7)
$70,000 to $99,999	53 (54.1)	41 (53.2)	20 (54.1)
$100,000 or more	3 (3.1)	2 (2.6)	0 (0)

Education			
Grade 12	10 (9.9)	9 (11.5)	4 (10.8)
Some postsecondary	22 (21.8)	15 (19.2)	4 (10.8)
Bachelor׳s degree	33 (32.7)	28 (35.9)	17 (45.9)
Graduate degree	21 (20.8)	14 (17.9)	9 (24.3)
Technical/trade school degree	15 (14.9)	12 (15.4)	3 (8.1)

Employment			
Unemployed	9 (8.9)	8 (10.3)	4 (10.8)
Part-time	22 (21.8)	15 (19.2)	11 (29.7)
Full-time	63 (62.4)	49 (62.8)	20 (54.1)
Stay-at-home mom	7 (6.9)	6 (7.7)	2 (5.4)

Type of IBD			
Crohn׳s disease	69 (68.3)	54 (69.2)	25 (67.6)
Ulcerative colitis	29 (28.7)	21 (26.9)	10 (27)
Indeterminate	3 (3.0)	3 (3.8)	2 (5.4)

Reproductive history			
Have biological children	26 (25.7)	19 (24.4)	8 (21.6)
Currently pregnant	11 (10.9)	5 (6.4)	1 (2.7)
Ever been pregnant	27 (26.7)	19 (24.4)	9 (24.3)

Medication history			
Sulfasalazine	12 (11.9)	10 (12.8)	6 (16.2)
Mesalamine/5-ASA	83 (82.2)	67 (85.9)	31 (83.8)
Budesonide	20 (19.8)	17 (21.8)	8 (21.6)
Steroids	82 (81.2)	63 (80.8)	31 (83.8)
Methotrexate	15 (14.9)	12 (15.4)	9 (24.3)
Azathioprine/mercaptopurine	72 (71.3)	55 (70.5)	27 (73)
Anti-TNF/biologics	54 (53.5)	42 (53.8)	22 (59.5)
Antibiotics	50 (49.5)	38 (48.7)	18 (48.6)
Study medications	10 (9.9)	8 (10.3)	4 (10.8)

Saw IBD specialist in outpatient gastroenterology clinic in the past year			
Yes	84 (84.0)	67 (85.9)	33 (89.2)
No	16 (16.0)	11 (14.1)	4 (10.8)

Hospitalized for IBD in the past year			
Yes	22 (22.)	16 (20.5)	4 (10.8)
No	78 (78.0)	62 (79.5)	33 (89.2)

Discussed pregnancy in IBD topics with:			
Gastroenterologist	46 (45.5)	36 (46.2)	19 (51.4)
General medicine specialist	2 (2.0)	2 (2.6)	0 (0)
Gynecologist/obstetrician	17 (16.8)	12 (15.4)	7 (18.9)
Family physician	22 (21.8)	18 (23.1)	7 (18.9)
IBD nurse	16 (15.8)	11 (14.1)	4 (10.8)
Pharmacist	1 (1.0)	1 (1.3)	0 (0.0)
Family and friends	36 (35.6)	29 (37.2)	12 (35.1)
Support groups	2 (2.0)	2 (2.6)	1 (2.7)

Obtained information regarding pregnancy in IBD from:			
Internet	52 (51.5)	42 (53.8)	22 (59.5)
Pamphlets and brochures	18 (17.8)	14 (17.9)	7 (18.9)
Books	6 (5.9)	4 (5.1)	1 (2.7)

Baseline CCPKnow level			
Poor (0–7)	49 (48.5)	37 (47.4)	16 (43.2)
Adequate (8–10)	27 (26.7)	22 (28.2)	9 (24.3)
Good (11–13)	15 (14.9)	13 (16.7)	8 (21.6)
Very good (14–17)	10 (9.9)	6 (7.7)	4 (10.8)

Baseline CCPKnow**—**dichotomized			
Poor (0–7)	49 (49.5)	37 (47.4)	16 (43.2)
Adequate+ (>7)	52 (51.5)	41 (52.6)	21 (56.8)
